# Effects of Gender, Menopause, Vitamin D Status, and Tumor Parathyroid Cell Activity on Serum Phosphate Levels in a Large Cohort of Patients with Sporadic Hypercalcemic Primary Hyperparathyroidism

**DOI:** 10.3390/ijms27042012

**Published:** 2026-02-20

**Authors:** Matteo Corbetta, Silvia Carrara, Anna Dal Lago, Romina Mirsepanj, Elena Ruotolo, Chiara Sardella, Giacomo De Leo, Filomena Cetani, Sabrina Corbetta

**Affiliations:** 1 Department of Clinical Sciences and Community, University of Milan, 20122 Milan, Italy; matteo.corbetta@unimi.it (M.C.); romina.mirsepanj@unimi.it (R.M.); elena.ruotolo@unimi.it (E.R.); 2 Bone Metabolism Diseases and Diabetes Unit, IRCCS Istituto Auxologico Italiano, 20145 Milan, Italy; silvia.carrara1@unimi.it; 3 Department of Clinical and Experimental Medicine, University of Pisa, 56124 Pisa, Italy; anna.dallago@unipi.it (A.D.L.); giacomo.deleo@unipi.it (G.D.L.); 4 Unit of Endocrinology, University Hospital of Pisa, 56124 Pisa, Italy; chiarasardella@virgilio.it; 5 Department of Biochemical, Surgical and Dental Sciences, University of Milan, 20122 Milan, Italy

**Keywords:** hypophosphatemia, primary hyperparathyroidism, hypercalcemia, PTH

## Abstract

Diagnosis of primary hyperparathyroidism (PHPT) relies on the detection of hypercalcemia and increased circulating parathormone (PTH) levels. However, the disease induces a deep deregulation of phosphate metabolism. A total of 960 PHPT patients (848 females, 112 males) were retrospectively enrolled; biochemical and clinical data were collected at PHPT diagnosis. At variance with previous studies, hypophosphatemia was diagnosed using sex- and age-specific serum phosphate reference range. Reduced serum phosphate levels were detectable in 49% of PHPT males and 55% of PHPT females. Moderate hypophosphatemia (≤2.0 mg/dL) was more frequent in men than in women, and serum phosphate levels were lower in postmenopausal than premenopausal PHPT women. Vitamin D status did not alter the prevalence of hypophosphatemia. Serum phosphate levels negatively correlated with ionized calcium and PTH levels across PHPT premenopausal women, postmenopausal women, and men. Cluster analysis integrating the three interrelated parameters identified three distinct PHPT phenotypes: bone and kidney complications were more frequent in patients with more severe hypercalcemia and hypophosphatemia, though fractures were more abundant in the less severe phenotypes. Finally, considering the whole cohort, ionized calcium and PTH levels displayed a negative non-linear correlation with phosphate levels. In conclusion, hypophosphatemia in PHPT patients is common, and moderate hypophosphatemia is more frequent in males compared to females. Menopausal status is associated with less severe hypophosphatemia and PHPT disease. Hypophosphatemia is mainly determined by parathyroid tumor cells’ dysfunction. The non-linear negative relationships between phosphate, PTH and ionized calcium may suggest heterogeneous insensitivity of tumor parathyroid cells to extracellular phosphate.

## 1. Introduction

Primary hyperparathyroidism (PHPT) is a common endocrine disorder characterized by autonomous parathyroid hormone (PTH) secretion, resulting in chronic dysregulation of calcium–phosphate homeostasis [1].

Under physiological conditions, PTH secretion is tightly regulated by serum calcium levels through activation of calcium-sensing receptor (CASR) [2]. Chronic low serum ionized calcium concentrations and hyperphosphatemia stimulate PTH secretion [3]. In addition, PTH exerts a phosphaturic effect through two main mechanisms: (1) inhibition of transcription of the sodium-dependent phosphate transport protein 2A (NaPi2a) and 2C (NaPi2c) in the proximal convoluted tubule, and (2) activation of intracellular signaling pathways, including protein kinase A, protein kinase C and MAPK, which promote rapid internalization and lysosomal degradation of NaPi2a and NaPi2c. These actions reduce renal phosphate reabsorption; consequently, phosphaturia increases [4,5]. It is widely accepted that PTH and phosphate levels are inversely correlated: higher PTH concentrations are associated with lower serum phosphate [6,7].

Hypophosphatemia is common in PHPT, with a prevalence ranging from 10 to 20% [8] up to 42% [9]. In the Indian PHPT population, the prevalence appeared higher, reaching 53%, likely due to a greater prevalence of vitamin D deficiency [10]. In addition, serum phosphate levels are reported to be lower in male PHPT patients [11] and in patients with kidney stones [12,13]. However, most patients with PHPT maintain normal phosphate levels due to compensatory increases in intestinal absorption and bone resorption [10].

Recently, it has been demonstrated that serum phosphate levels are characterized by gender difference and age-related declining in the general adult population [14,15]. Therefore, when evaluating the prevalence and effects of hypophosphatemia in PHPT patients, the use of gender- and age-specific reference intervals may play a significant role.

Hypercalcemia is traditionally considered the hallmark biochemical feature of PHPT. Meanwhile, serum phosphate levels are poorly considered in PHPT diagnosis or management. Serum and urinary phosphate measurements are not required for diagnosis, and they are not included among the criteria for surgical intervention. Current PHPT management guidelines do not provide phosphate cut-offs, and no recommendations are based on this parameter [16,17].

The present study assessed the following hypothesis in a large PHPT cohort: (1) prevalence of hypophosphatemia may vary considering specific gender-related reference interval; (2) gender, menopause, and vitamin D status may affect hypophosphatemia; (3) serum phosphate levels may relate with clinical and hormonal features in PHPT patients.

## 2. Results

### 2.1. Prevalence of Hypophosphatemia in PHPT Patients

A total of 960 patients (848 females, 112 males) diagnosed with hypercalcemic PHPT were enrolled, of whom 410 underwent parathyroidectomy.

1. Effect of gender. In the present PHPT cohort, women exhibited higher median serum phosphate levels than those detected in men (2.70 vs. 2.35 mg/dL; *p* < 0.001). Referring to the established lower limit of the reference interval, namely 2.32 mg/dL for males and 2.73 mg/dL for females [14,15], hypophosphatemia was diagnosed in 55 (49%) PHPT men and in 467 (55%) PHPT women (*p* = 0.267). Among hypophosphatemic patients, moderate hypophosphatemia, defined as serum phosphate levels ≤ 2.0 mg/dL, was observed in 52.7% of men and 17.3% of women. No patients of either sex had serum phosphate levels ≤ 1.0 mg/dL. Overall, male PHPT patients displayed lower serum phosphate levels than females.
2. Effects of menopause status. Premenopausal PHPT women had lower median phosphate levels than those detected in postmenopausal women (Table 1). Moderate hypophosphatemia occurred in 12.2% of premenopausal women and in 9.0% of postmenopausal women. Notably, urine phosphate excretion levels were higher in premenopausal than in postmenopausal women (Table 1). These findings suggest that estrogen deficiency is associated with less pronounced alterations of phosphate metabolism.
3. Effect of vitamin D repletion. Among PHPT patients with vitamin D repletion (n = 447), defined as serum 25-hydroxyvitamin D (25OHD) > 30 ng/mL according to current PHPT management guidelines [16,17], reduced serum phosphate levels were diagnosed in 52.5% of women and 47.0% of men. Moderate hypophosphatemia (≤2.0 mg/dL) occurred in 46.7% of vitamin D-repleted PHPT men and in 12.8% of vitamin D-repleted PHPT women, indicating that vitamin D status marginally affected serum phosphate levels.

### 2.2. Correlations of Serum Phosphate Levels with Biochemical and Clinical Features According to Gender and Menopausal Status

Serum phosphate levels negatively correlated with ionized calcium and PTH_ULN levels among pre- and postmenopausal women as well as men (Table 2). Though any correlation could be detected between serum phosphate and 24 h urine phosphate levels, they negatively correlated with 24 h urine calcium excretion in both pre- and postmenopausal women, but not in men. Of note, in postmenopausal women, serum phosphate levels positively correlated with 25OHD levels. Significant correlation with any other clinical or biochemical parameters could not be detected, namely age at diagnosis; BMI; eGFR; lumbar spine, femur, neck, and total hip T-scores; and number of fragility fractures. Indeed, cross-sectional correlations may not be sensitive in identifying correlations with skeletal outcomes.

### 2.3. Phenotypes of PHPT Patients According to the Mineral Metabolic Profile

Since serum phosphate levels significantly correlated across premenopausal women, postmenopausal women, and men only with ionized calcium and PTH levels, hierarchical clustering was performed using serum phosphate levels, ionized calcium levels and PTH_ULN to identify PHPT phenotypes associated with different mineral metabolic profiles. The analysis identified three distinct phenotypes (Table 3), whose patients did not differ in age at diagnosis as well as BMI.

Phenotype 1 included PHPT patients with more severe clinical features, who exhibited lower hypophosphatemia, higher hypercalcemia and higher PTH levels, higher prevalence of kidney complications, namely reduced eGFR, CKD (26.1%), and kidney stones (45.2%), more severe osteoporosis at lumbar and femur sites, higher prevalence of arterial blood hypertension (52.0%). Phenotype 1 was characterized by vitamin D insufficiency with median 25OHD levels of 20.1 ng/mL; 19.5% of phenotype 1 PHPT patients were diagnosed with severe vitamin D deficiency (25OHD < 12.0 ng/mL). Of note, in phenotype 1, fragility fractures tended to be less frequent, while cardiovascular- and cerebrovascular events tended to be more prevalent (12.4%). These features may be related to the higher proportion of included males. Serum phosphate levels did not correlate with 24 h urine phosphate excretion (r = −0.067, *p* = 0.393 by Spearman’s correlation).

Phenotype 2 included PHPT patients with the milder clinical features, showing higher phosphate levels, lower serum ionized- and albumin-corrected calcium levels, lower PTH levels, higher eGFR, lower urine calcium excretion, lower prevalences of kidney stones (32.8%), CKD (9.6%), hypertension (39.8%), and lower prevalence of cardiovascular and cerebrovascular events (5.7%). In these patients, serum phosphate levels negatively correlated with 24 h urine phosphate excretion (r = −0.127, *p* = 0.033 by Spearman’s correlation).

Phenotype 3 included patients with intermediate biochemical features, while the lumbar spine (LS), femur neck (FN) and total hip (TH) T-scores, as well as the prevalence of fragility fractures, were similar to those in patients displaying phenotype 2. Similarly to patients with phenotype 2, serum phosphate levels negatively correlated with 24 h urine phosphate excretion (r = −0.202, *p* = 0.014 by Spearman’s correlation).

The analysis of the phosphate-related phenotypes suggested that levels of phosphate reflect the severity of the PHPT disease.

However, multiple linear regression analysis showed that phosphate levels were predicted only by ionized calcium levels in phenotypes 1 and 3, while they were predicted by BMI, PTH and ionized calcium in phenotype 2.

### 2.4. Correlation of Serum Phosphate Levels with PTH and Ionized Calcium in the Whole PHPT Cohort

**Figure 1:**
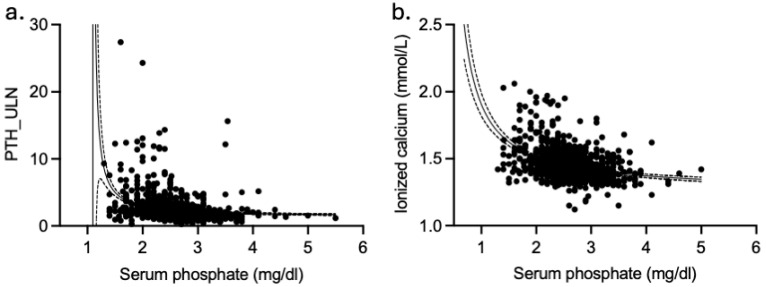
Relationship between serum phosphate levels and PTH levels (**a**) and ionized calcium levels (**b**). Dash lines represent 95% confidence interval. PTH_ULN, parathyroid hormone as folds of the upper limit of the reference interval.

Considering that serum phosphate levels emerge as mainly determined by ionized calcium across the identified PHPT phenotypes, we focused the attention on its relationship with ionized calcium and PTH levels in the whole cohort of PHPT patients. Serum phosphate levels showed a negative correlation with both ionized calcium and PTH_ULN levels (Figure 1a,b). The negative correlations were confirmed, but the best fitting curve was non-linear second order quadratic line: little changes in ionized calcium (adjusted r^2^ = 0.165, Sx·y = 0.112) and PTH levels (adjusted r^2^ = 0.102, Sx·y = 2.017) were associated with wide changes in serum phosphate levels. The magnitude of Sx.y suggests that the selected model provides an adequate and biologically plausible description of the phosphate-ionized calcium/PTH relationship. Moreover, serum phosphate levels negatively correlated with 24 h urine calcium excretion levels (n = 837, r = −0.233, *p* < 0.001 by Spearman’s correlation test). The non-linear relationship suggests that other factors may be involved in phosphate metabolism in PHPT disease.

## 3. Discussion

Hypophosphatemia, often overlooked in the diagnostic work-up of patients with PHPT, has recently received attention. Although hyperparathyroidism is expected to be associated with hypophosphatemia due to the phosphaturic effects of PTH, hypophosphatemia is not observed in all PHPT patients. The reported prevalence of hypophosphatemia in PHPT patients varies considerably across the different series. These heterogenous results reflects at least in part differences in laboratory reference intervals for serum phosphate. Most laboratories apply the same reference ranges for adult men and women, despite growing evidence for age- and sex-related differences. Recently, in the general Caucasian adult population aged more than 45 years from the Rotterdam study [14,15], serum phosphate levels were shown to differ by gender, being lower in males than in females, and by aging-related declining, in a sex-independent manner. Furthermore, investigation of sex differences in mineral disorders has been identified as a key priority in the evidence-based roadmap developed by the EndoCompass project for calcium and bone endocrinology [18]. Accordingly, in the present study, we analyzed a large cohort of Italian PHPT patients referred to third-level endocrine centers. Using a sex- and age-specific serum phosphate refence range [14], reduced serum phosphate levels emerge as a mineral metabolic abnormality detectable in approximately half of PHPT patients, specifically, 49% of men and 55% of women. However, when moderate hypophosphatemia, defined as serum phosphate levels ≤ 2.0 mg/dL, was considered, it was detected in about half of hypophosphatemic PHPT men, but only in 17% of hypophosphatemic PHPT women. Severe, life-threatening hypophosphatemia lower than 1.0 mg/dL was not observed.

Hormonal changes associated with menopause also influence serum phosphate levels. Estradiol has been shown to lower phosphate levels in the general population [15], with serum estradiol inversely associated with serum phosphate in both sexes. Serum testosterone shows an inverse association with serum phosphate in both sexes with an apparent stronger effect in men than in women [15]. In our cohort, this estrogenic effect was evident, as premenopausal women exhibited significant lower serum phosphate levels than postmenopausal women. Nevertheless, the prevalence of moderate hypophosphatemia did not differ between the two groups. These findings are consistent with previous reports [9], highlighting the need for considering gender in the evaluation of the entity of hypophosphatemia in PHPT. Notably, detection of hypophosphatemia ≤ 2.0 mg/dL in postmenopausal women should be regarded as a marker of severe PHPT disease.

At variance with the concept that vitamin D deficiency exacerbates hypophosphatemia [10], in the present study, the prevalence of hypophosphatemia in vitamin D-repleted females and males did not differ from that detected in the overall cohort. Correspondingly, the proportion of moderate hypophosphatemia in hypophosphatemic vitamin D-repleted PHPT patients was also similar. However, in postmenopausal women, whose median 25OHD levels exceeded 30 ng/mL, serum phosphate levels showed a positive correlation with serum 25OHD levels. This finding suggests that in this setting of PHPT women, supplementation with cholecalciferol may help in controlling PTH secretion from tumor parathyroid cells, without significantly worsening hypercalcemia or hypophosphatemia. Supporting this observation, a prospective randomized controlled study in 70 PHPT patients and 75 controls treated with weekly 14,000 IU of vitamin D3, demonstrated that supplementation was safe and efficient to increase serum 25OHD levels in both groups [19]. The Authors described a paradoxical reduction in serum 1,25OH_2_D levels, and a rise in fibroblast growth factor 23 (FGF23) levels in both groups, possibly representing a protective response against hypercalcemia [19].

The present study showed that across PHPT premenopausal women, postmenopausal women, and men, serum phosphate levels constantly correlated only with ionized calcium and PTH levels. Therefore, integration of phosphate levels with the biomarkers ionized calcium and PTH classically used for PHPT diagnosis, allowed identification of three distinct PHPT phenotypes. Phenotype 1 was characterized by more severe biochemical abnormalities compared with the remaining two groups, with lower median phosphate levels, higher prevalence of bone and kidney complications, namely osteoporosis at lumbar and femur sites, kidney stones and CKD, while fractures tended to be less prevalent. The lower prevalence of fractures should be considered with caution as phenotype 1 included a higher proportion of PHPT males, in whom the fracture risk related to postmenopausal estrogen deficiency is absent. Phenotype 2 exhibited milder biochemical disturbances, namely milder hypercalcemia and hypophosphatemia, fewer kidney complications, and a predominance of osteopenia, though fractures at diagnosis were about twice more than those detected among phenotype 1 patients. Phenotype 3 displayed intermediate characteristics.

Phenotype 2 closely resembles the clinical profile associated with downregulation of both *CASR* and vitamin D receptor (*VDR*) transcripts, which has been previously identified through gene expression profiling of a set of parathyroid-related genes in a series of parathyroid adenomas from sporadic PHPT patients [20]. Emerging evidence indicates that CASR also functions as a phosphate sensor. Extracellular phosphate acts as a partial, non-competitive antagonist of CASR, thereby modulating PTH secretion, while negatively charged phosphate can reduce CASR activity both extracellularly—through interactions with arginine residues—and intracellularly, via covalent phosphorylation [21,22]. In agreement with previous studies, the present results confirmed the inverse relationship between serum phosphate and PTH levels in PHPT patients. However, our data revealed that ionized calcium and PTH levels are the main determinants of serum phosphate levels in the PHPT setting, indicating that serum phosphate levels are largely driven by parathyroid tumor cells’ activity. At the molecular level, both ionized calcium and PTH levels are modulated by CASR according to a sigmoidal relationship. The sigmoid curve is typically right-shifted in parathyroid tumor cells from PHPT patients [2,23].

The absence of a linear relationship between serum phosphate and both PTH and ionized calcium levels highlights the profound dysregulation of the mineral metabolism in PHPT and suggests the involvement of additional metabolic regulatory pathways. These include (1) increased synthesis of 1,25OH_2_D, which may enhance the intestinal absorption of phosphate; (2) elevated FGF23 levels, commonly observed in PHPT patients, which can exacerbate hypophosphatemia by promoting urine phosphate excretion. FGF23 showed a positive correlation with calcium in PHPT patients [24], and extracellular calcium can increase FGF23 levels independently of vitamin D and PTH, though part of the physiological increase in FGF23 induced by extracellular calcium is mediated by vitamin D signaling [25]. However, it should be kept in mind that parathyroid tumor cells are often characterized by insensitivity to extracellular calcium as well as 1,25OH_2_D; (3) CASR-mediated effects on phosphate handling, as CASR regulates both PTH and FGF23 secretion and may directly influence renal phosphate transport. It has been demonstrated that CASR represents a phosphate sensor in the parathyroid gland, explaining the stimulatory effect of phosphate on PTH secretion in physiological condition [21,22]; (4) dietary phosphate intake and/or intestinal absorption; phosphaturia and phosphate transporter regulation occur even in the absence of PTH and FGF23 signaling. Dietary intake of phosphate and the phosphate intestinal absorption rate might act as modulators of the PHPT phenotypes, resembling the known effects of dietary calcium intake and intestinal calcium absorption [26].

Finally, analysis of the relationship between serum phosphate and urinary phosphate excretion revealed phenotype-specific patterns. In patients with phenotypes 2 and 3, serum phosphate levels inversely correlated with urine phosphate excretion, whereas no significant correlation was observed in phenotype 1. This finding suggests that the phosphate derangement associated with severe deregulated parathyroid tumor activity, may involve pathophysiological mechanisms extending beyond PTH hypersecretion alone. Further studies are warranted to elucidate the molecular basis of the insensitivity to phosphate in PHPT, as these mechanisms may represent novel therapeutic targets. In this context, investigating phosphate regulation in normocalcemic PHPT could provide insight into intermediate disease phenotypes.

The strengths of the present study include the large, contemporary PHPT cohort, and the standardized clinical evaluation across participating centers. Nevertheless, several limitations should be acknowledged. First, the retrospective design inherently limits causal inference. Second, despite participating centers followed standardized diagnostic criteria, variability in assay platforms and local clinical practices cannot be entirely excluded. Third, key regulators of phosphate metabolism—such as FGF23 and 1,25-dihydroxyvitamin D—were not systematically measured. Additionally, bone turnover markers evaluation, as bone is the major deposit of phosphate, and, similarly, tubular maximal resorption of phosphate (TmP), were not available.

Future studies should also systematically evaluate the clinical effects of hypophosphatemia on bone, skeletal muscle and kidney functions to define the direct effect of hypophosphatemia in determining the clinical complications. Although hypophosphatemia is common in PHPT patients, at present there is no recommendation about its management. Oral phosphate and calcitriol supplementation should be avoided due to further stimulation of PTH secretion and worsening of serum- and urine calcium levels [27]; in addition, cholecalciferol supplementation in PHPT patients with concomitant hypovitaminosis D is recommended as it is generally safe and may lower PTH levels without affecting serum- and urine calcium levels [28].

## 4. Materials and Methods

PHPT was diagnosed detecting concomitant increased ionized and/or albumin-adjusted serum calcium and increased or inappropriately normal intact PTH levels [16,17]. All patients were evaluated at three tertiary care referral Italian Endocrine Centers for the management of bone and mineral diseases during the period 2015–2025: AO Universitaria Pisana in Pisa (n = 311), IRCCS Istituto Ortopedico Galeazzi (in the period 2015–2023) and IRCCS Istituto Auxologico Italiano (in the period 2023–2025) in Milan (n = 649).

The following inclusion criteria were considered: (1) age ≥ 18 years; (2) individuals with symptomatic and asymptomatic PHPT according to recent guidelines [16,17]; and (3) hypercalcemia, defined as plasma ionized calcium > 1.32 mmol/L and/or serum albumin-corrected calcium > 10.4 mg/dL in at least two distinct occasions.

Exclusion criteria were as follows: (1) age < 18 years; (2) clinical and/or genetic diagnosis of familial hypocalciuric hypercalcemia (HHC, OMIM 145980, 145981, and 600740); (3) familial PHPT (MEN1, OMIM 131100; MEN2, OMIM 171400; MEN4, OMIM 610755; HPT-JT, and OMIM145001); (4) parathyroid carcinoma; (5) ionized calcium levels < 1.32 mmol/L; (6) diseases (with the exception of diabetes) or therapies affecting bone metabolism; (7) ongoing (previous 6 months) antiresorptive treatment; (8) lack of DXA measurement at lumbar or hip sites; and (9) DXA not performed by a Hologic or Lunar densitometer.

### 4.1. Study Design

This is a retrospective study considering data collected at the time of PHPT diagnosis; each enrolled patient underwent standard evaluation according the most current guidelines and the following data were collected: (1) circulating ionized and albumin-corrected calcium, phosphate, creatinine, 25OHD, and PTH; (2) 24 h urinary calcium measurement; (3) anthropometric data (weight and height) and calculation of body mass index (BMI); (4) bone mineral density (BMD) assessment by DXA (Hologic or Lunar) at lumbar and total hip and femoral neck; (5) clinical and morphometric fractures detected by conventional thoracic and lumbar X-rays; (6) previous hip, pelvic, ankle, humerus, and wrist fragility fractures; (7) concomitant comorbidities (diabetes, arterial blood hypertension, and others); and (8) occurrence of symptomatic or asymptomatic kidney stones.

All procedures performed in the present study involving human participants were in accordance with the ethical standards of the institutional and/or national research committee and with the 1964 Helsinki Declaration and its later amendments or comparable ethical standards. No ethic committee approval was required according to the Italian law given the observational retrospective design of the study [Ministero della Salute, Attività dei Comitati Etici Istituiti ai Sensi del Decreto Ministeriale 18 Marzo 1998. Circolare Ministeriale n.6 del 2 Settembre 2002. G.U. n. 214 12 Settembre 2002. https://www.aosp.bo.it/sites/default/files/2002-09-02_0.pdf (Accessed on 19 October 2022)]. All the procedures performed were part of the routine care. All patients gave their informed consent, and data were anonymously collected in a database.

### 4.2. Biochemistry

Blood samples for biochemical and hormonal determinations were withdrawn after overnight fasting and rest. The collection of 24 h urine was obtained under free diet conditions. Serum total calcium, albumin, phosphate, creatinine, and 24 h urinary calcium and phosphate were analyzed by a standard autoanalyzer using colorimetric and enzymatic methods. Ionized serum calcium was analyzed by an ion-selective electrode method after correction for the pH. Plasma PTH was measured by a second- (IRCCS Ospedale Galeazzi Sant’Ambrogio and IRCCS Istituto Auxologico Italiano) and third-generation assay (AO Universitaria Pisana). Thus, PTH levels were treated as fold change in the upper limit of the normal range for each specific assay (PTH_ULN). Serum 25OHD was measured by chemiluminescent immunoassay. Calcium and phosphate excretions were routinely measured in 24 h urine collection. The estimated glomerular filtration rate (eGFR) was calculated using the EPI-CKD (Chronic Kidney Disease Epidemiology Collaboration) formula [29].

### 4.3. Bone Mineral Density Measurements

DXA QDR-4500 (Hologic, Bedford, MA, USA) or Lunar iDXA (GE Healthcare, GE Medical System Italia, milan, Italy) were used to measured BMD at the lumbar spine (L1–L4), proximal femur, and total hip. BMD was expressed as T-scores (difference from the mean BMD value of healthy young people in SD units). According to World Health Organization (WHO) recommendations, osteoporosis and osteopenia were defined as a T-score value ≤ −2.5 SD and <−1 and >−2.5, respectively.

### 4.4. Fracture Assessment

The presence of VFs was assessed in all patients at diagnosis by conventional thoracic and lumbar X-rays at the Milan Units, while at the Pisa Unit, VFs were assessed by DXA morphometry and, when positive, confirmed by X-rays. Vertebrae were classified as normal (<25% reduction) or fractured (>25% reduction in vertebral body height, i.e., moderate deformity according the Genant semiquantitative grading scale for VFs) [30]. X-rays were graded by visual inspection of two trained reviewers at each center. Major low-trauma fractures (femur, proximal humerus, or wrist) were recorded in all subjects by medical history.

### 4.5. Statistical Analysis

Data were collected from clinical records. Nonparametric data failing normality test was expressed as median and interquartile range; normally distributed data were expressed as mean ± standard deviation. Comparison among proportions were performed using Fischer’s exact test. Data were analyzed by Spearman’s correlation test corrected for multiple correlations to identify parameters significantly correlated with serum phosphate levels. Multiple regression analysis using a least squares model including ionized calcium; PTH_ULN; phosphate; and femur, neck, and total hip T-scores, considering serum phosphate levels as dependent variable and ionized calcium, PTH_ULN, age at diagnosis, number of fractures, lumbar and femur T-scores, as independent variables, indicated that phosphatemia were predicted by ionized calcium and PTH_ULN; all other variables were not predictive. Serum phosphate, ionized calcium, and PTH_ULN levels were considered as criteria for performing unsupervised hierarchical clusterization by using Wards’ method and Euclidean similarity index. The method highlights natural patterns in the data without a priori assumptions; three clusters were selected based on the dendrogram structure, corresponding to the largest increase in within-cluster variance, and because this solution provided stable and biologically interpretable groups. Data were compared among the three identified clusters by Kruskal–Wallis ANOVA analysis corrected for multiple comparisons (Dunn’s test).

Statistical analysis was performed by GraphPad Prism version 10.6.1 for Mac OS X, GraphPad Software, San Diego, California USA, www.graphpad.com. A value of *p* less than 0.05 was considered as statistically significant.

## 5. Conclusions

Hypophosphatemia in PHPT patients is common, and more frequent in male patients than females. Menopausal status is associated with less severe hypophosphatemia and PHPT disease. Hypophosphatemia is mainly determined by parathyroid dysfunction, though the negative relationship with PTH and ionized calcium lacks linearity in PHPT patients, suggesting partial heterogeneous insensitivity of tumor parathyroid cells to extracellular phosphate. Phosphate metabolism should be more deeply investigated in PHPT patients, including the assessment of all the modulators, namely FGF23, 1,25OH_2_D, TmP, and bone turnover markers, to clarify the role of the deregulated phosphate metabolism in PHPT clinical complications in each patient.

## Figures and Tables

**Table 1:** Comparisons of biochemical and clinical features among PHPT premenopausal women, postmenopausal women, and men.

Parameter	Premenopausal Women	Postmenopausal Women	Men	*p*
n	147	700	113	
Age (years)	49.0 (43.0, 52.0)	65.0 (59.0, 74.0) *	66.5 (55.0, 74.0) *	<0.001
BMI (kg/m^2^)	23.4 (20.3, 28.4)	25.4 (22.3, 29.6) *	26.2 (24.3, 28.0) *	0.007
Ca^2+^ (mmol/L)	1.45 (1.39, 1.53)	1.42 (1.39, 1.49) *	1.45 (1.39, 1.54) §	0.002
Alb-corr Ca (mg/dL)	10.9 (10.5, 11.4)	10.8 (10.5, 11.3)	11.1 (10.7, 11.7) §	0.001
Serum P (mg/dL)	2.6 (2.2, 2.9)	2.7 (2.4, 3.0) *	2.3 (2.0, 2.6) *§	<0.001
Moderate hypoP (%)	12.2	9.0	52.7 *§	<0.001
PTH_ULN	2.05 (1.32, 3.00)	1.80 (1.40, 2.53)	2.13 (1.58, 3.26) §	0.003
25OHD (ng/mL)	26.7 (19.8, 35.8)	32.0 (25.7, 41.0) *	26.0 (15.7, 35.2) §	<0.001
eGFR (mL/min)	100.2 (85.6, 106.8)	85.8 (71.5, 94.5) *	77.1 (60.6, 90.7) *§	<0.001
UCa (mg/24 h)	375.0 (273.5, 475.5)	281.0 (196.0, 400.0) *	350.0 (221.5, 470.5) §	<0.001
UP (mg/24 h)	756.0 (627.0, 954.0)	700.0 (558.0, 870.0) *	900.0 (687.5, 1089.0) §	<0.001

*, *p* < 0.05 vs. premenopausal women; §, *p* < 0.05 vs. postmenopausal women; BMI, body mass index; Ca^2+^, ionized calcium; Alb-corr Ca, serum albumin-corrected calcium; P, phosphate; moderate hypoP, moderate hypophosphatemia, ≤2.0 mg/dL; PTH_ULN, parathyroid hormone as folds of the upper limit of the reference interval; 25OHD, 25-hydroxyvitamin D; eGFR, estimated glomerular filtration rate; UCa, 24 h urine calcium excretion; UP, 24 h urine phosphate excretion.

**Table 2:** Correlations between serum phosphate levels and biochemical and clinical parameters in premenopausal women, postmenopausal women, and men. Data were analyzed by non-parametric Spearman’s correlation matrix.

Parameter	Premenopausal Women	Postmenopausal Women	Men
BMI	−0.164, *p* = 0.096	−0.144, *p* = 0.011	−0.015, *p* = 0.907
Ca^2+^	−0.426, *p* < 0.001	−0.406, *p* < 0.001	−0.291, *p* = 0.004
Alb-corr Ca	−0.297, *p* < 0.001	−0.342, *p* < 0.001	−0.126, *p* = 0.185
PTH_ULN	−0.505, *p* < 0.001	−0.396, *p* < 0.001	−0.208, *p* = 0.027
25OHD	0.107, *p* = 0.263	0.176, *p* < 0.001	0.045, *p* = 0.670
UCa	−0.286, *p* = 0.001	−0.213, *p* < 0.001	−0.118, *p* = 0.260

BMI, body mass index; Ca^2+^, ionized calcium; Alb-corr Ca, serum albumin-corrected calcium; PTH_ULN, parathyroid hormone as folds of the upper limit of the reference interval; 25OHD, serum 25-hydroxyvitamin D; UCa, 24 h urine calcium excretion.

**Table 3:** Comparisons of biochemical and clinical features among the different phenotypes of PHPT patients.

Parameters	Phenotype 1	Phenotype 2	Phenotype 3	*p*
n	111	537	312	
Age (years)	67.0 (54.0, 76.0)	63.0 (56.0, 71.0)	63.0 (55.0, 73.0)	0.263
Males (%)	18.9	9.3 *	13.1	0.011
BMI (kg/m^2^)	25.8 (22.2, 29.6)	25.1 (22.4, 29.0)	25.0 (22.1, 28.8)	0.705
Ca^2+^ (mmol/L)	1.58 (1.46, 1.73)	1.39 (1.36, 1.45) *	1.47 (1.40, 1.55) *§	<0.001
Alb-corr Ca (mg/dL)	11.8 (11.3, 12.9)	10.7 (10.4, 11.0) *	11.1 (10.7, 11.6) *§	<0.001
Serum P (mg/dL)	2.2 (1.9, 2.5)	2.8 (2.5, 3.1) *	2.5 (2.3, 2.8) *§	<0.001
PTH_ULN	5.17 (4.41, 7.40)	1.45 (1.16, 1.68) *	2.51 (2.21, 2.93) *§	<0.001
25OHD (ng/mL)	20.1 (13.8, 31.0)	33.0 (26.0, 42.0) *	30.0 (22.2, 37.0) *§	<0.001
eGFR (ml/min)	78.1 (60.0, 95.0)	88.4 (75.4, 96.0) *	84.0 (66.1, 96.4)	0.004
UCa (mg/24 h)	371.0 (227.0, 526.6)	283.0 (195.0, 400.0) *	322.0 (230.0, 433.5) §	<0.001
UP (mg/24 h)	693.0 (500.0, 909.0)	702.0 (570.0, 900.0)	783.0 (600.0, 900.0)	0.093
LS T-score	−2.5 (−3.3, −1.6)	−2.3 (−3.1, −1.2)	−2.2 (−3.1, −1.1)	0.213
FN T-score	−2.5 (−3.2, −1.7)	−2.2 (−2.7, −1.5) *	−2.1 (−2.7, −1.4) *	0.004
TH T-score	−2.1 (−2.7, −1.2)	−1.7 (−2.3, −1.1) *	−1.7 (−2.4, −0.8) *	0.018
Fragility fractures (n)	0.28 ± 0.91	0.50 ± 1.16	0.48 ± 1.18	0.051
Kidney stones (%)	45.2	32.8 *	36.9	0.046
CKD (%)	26.1	9.6 *	11.6 *	<0.001
Hypertension (%)	52.0	39.8 *	46.1	0.036
Diabetes (%)	10.4	7.6	6.3	0.374
Dyslipidemia (%)	32.2	33.8	36.2	0.715
CVD (%)	12.4	5.7 *	6.9	0.054

*, *p* < 0.05 vs. cluster 1; §, *p* < 0.05 vs. cluster 2; BMI, body mass index; Ca^2+^, ionized calcium; Alb-corr Ca, albumin-corrected calcium; serum P, serum phosphate; PTH_ULN, parathyroid hormone as folds of the upper limit of the reference interval; 25OHD, serum 25-hydroxyvitamin D; eGFR, estimated glomerular filtration rate; UCa, 24 h urine calcium excretion; UP, 24 h urine phosphate excretion; LS, lumbar spine; FN, femur neck; TH, total hip; CKD, chronic kidney diseases, defined as eGFR < 60 mL/min in at least two determinations [16]; hypertension, arterial blood hypertension; CVD, cardiovascular and cerebrovascular diseases.

## Data Availability

The data that support the findings of this study are openly available in “zenodo” at https://doi.org/10.5281/zenodo.18120973.

## Abbreviations

The following abbreviations are used in this manuscript:

PHPT	primary hyperparathyroidism
PTH	parathormone
25OHD	25 hydroxyvitamin D
1,25OH_2_D	1,25 dihydroxyvitamin D
eGFR	estimated glomerular filtration rate
FGF23	fibroblast growth factor 23
CKD	chronic kidney disease
CASR	calcium-sensing receptor
